# MiR-138-5p Targets MACF1 to Aggravate Aging-related Bone Loss

**DOI:** 10.7150/ijbs.71411

**Published:** 2022-07-18

**Authors:** Zhihao Chen, Ying Huai, Gaoyang Chen, Shuyu Liu, Yan Zhang, Dijie Li, Fan Zhao, Xiaofeng Chen, Wenjing Mao, Xuehao Wang, Chong Yin, Chaofei Yang, Xia Xu, Kang Ru, Xiaoni Deng, Lifang Hu, Yu Li, Songlin Peng, Ge Zhang, Xiao Lin, Airong Qian

**Affiliations:** 1 Lab for Bone Metabolism, Xi'an Key Laboratory of Special Medicine and Health Engineering; Key Lab for Space Biosciences and Biotechnology, Research Center for Special Medicine and Health Systems Engineering, NPU-UAB Joint Laboratory for Bone Metabolism, School of Life Sciences, Northwestern Polytechnical University, Xi'an, Shaanxi, 710072, China.; 2 Department of Spine Surgery, Shenzhen People's Hospital, Southern University of Science and Technology, Jinan University School of Medicine, Shenzhen, 518020, China.; 3 Key Laboratory of Biomedical Information Engineering of Ministry of Education, and Institute of Molecular Genetics, School of Life Science and Technology, Xi'an Jiaotong University, Xi'an, Shaanxi, 710049, China.; 4 Law Sau Fai Institute for Advancing Translational Medicine in Bone and Joint Diseases, School of Chinese Medicine, Hong Kong Baptist University, Hong Kong, SAR, 999077, China.; 5 Department of Gynaecology and Obstetrics, Xijing Hospital, Fourth Military Medical University, Xi'an, Shaanxi, 710032, China.

**Keywords:** senile osteoporosis, miR-138-5p, MACF1, osteoblast differentiation, aging-related bone loss

## Abstract

Senile osteoporosis is one of the major health problems in an aging society. Decreased bone formation due to osteoblast dysfunction may be one of the causes of aging-related bone loss. With increasing evidence suggesting that multiple microRNAs (miRNAs) play important roles in osteoblast function, the relationship between miRNAs and senile osteoporosis has become a popular research topic. Previously, we confirmed that mechanoresponsive miR-138-5p negatively regulated bone anabolic action. In this study, the miR-138-5p level was found to be negatively correlated with BMD and osteogenic markers in bone specimens of senile osteoporotic patients by bioinformatic analysis and experimental verification. Furthermore, high miR-138-5p levels aggravated the decrease of aged osteoblast differentiation* in vitro* and led to worse bone loss in aged osteoblastic miR-138-5p transgenic mice *in vivo*. We also previously identified that the target of miR-138-5p, microtubule actin cross-linking factor 1 (MACF1), could attenuate senile osteoporosis. Here, miR-138-5p was demonstrated to regulate aged osteoblast differentiation by targeting MACF1. Finally, the therapeutic inhibition of miR-138-5p counteracted the decrease in bone formation and aging-related bone loss in aged mice. Overall, our results highlight the crucial roles and the molecular mechanism of miR-138-5p in aging-related bone loss and may provide a powerful therapeutic target for ameliorating senile osteoporosis.

## Introduction

Senile osteoporosis is a degenerative disease and has become one of the major health problems worldwide with the demographic shifts caused by the dramatic increase in human life expectancy 1, 2. Only 31-36% of people above the age of 70 have normal bone density, while the remainder suffer from some form of osteopenia or osteoporosis, which is a major contributor to the loss of independence due to bone fractures in the elderly 3. Substantial changes in bone structure occur with aging, including decreases in the thickness and mass of trabecular bone, loss rather than sparing of cortical bone, and an increase in bone marrow fat 4. With aging, bone loss occurs due to a process in which the bone remodeling balance is biased toward bone resorption rather than bone formation due to the decrease in the number and activity of osteoblasts 5. The changes in aging bone that lead to osteoporosis are mediated at multiple levels, including hormonal changes, bone unloading, and the accumulation of senescent cells 4.

MicroRNAs (miRNAs) are small noncoding RNAs that play important roles in posttranscriptional gene regulation 6. Accumulating evidence has suggested that multiple miRNAs play essential regulatory roles in aged osteoblast differentiation and aging-related bone loss 7-10. Recently, miR-138-5p was reported to play vital roles in osteogenic differentiation and bone formation 11-13. In our previous studies, we have confirmed that miR-138-5p, as a mechanoresponsive miRNA, accounts for the mechanosensitivity of the bone anabolic response 14. In addition, miR-138-5p has been demonstrated to negatively regulate osteoblast differentiation by inhibiting β-catenin under simulated microgravity 15. However, the relationship between miR-138-5p and senile bone loss has not been investigated, and the underlying mechanisms need to be studied.

In this study, we identified miR-138-5p as a novel aging-related miRNA in humans, mice and cultured cells for the first time. Moreover, the miR-138-5p level was negatively correlated with osteoblast differentiation and bone formation. We subsequently validated the relationship of miR-138-5p with the target gene microtubule actin cross-linking factor 1 (MACF1) in aged osteoblast differentiation and senile osteoporosis by loss- and gain-of-function of miR-138-5p *in vitro*. Moreover, we verified the involvement of miR-138-5p in senile osteoporosis *in vivo* by comparing young and aged mice with osteoblastic miR-138-5p transgenic (TG) mice. We observed that aged TG mice showed more severe osteoporosis symptoms. Finally, the increased bone formation and reduced bone loss induced by administering miR-138-5p inhibitor to aged mice also suggested that the inhibition of miR-138-5p may be a potential therapeutic strategy for senile osteoporosis.

## Materials and Methods

### MicroRNA expression profile analysis of differentially expressed miRNAs in plasma of osteoporotic patients

The microRNA expression profile was tested in plasma of healthy patients (*T*-score ≥ -1, n = 6) or osteoporotic patients (*T*-score ≤ -2.5, n = 6) 16, 17. In this study, we further used this expression profile to analyze the relationship of miR-138-5p expression and bone mineral density (BMD) in osteoporotic patients. The microRNA expression profile has been uploaded to GEO database (GSE93883).

### Human bone specimen collection

The bone specimens of osteoporotic patients (11 female between 60 and 79 years of age were recruited) were collected from the Shenzhen People's Hospital (Table S1 and Table S2). The patients with malignancy, osteoarthritis or systemic inflammatory diseases, liver and kidney diseases, chronic corticosteroid use, or other severe diseases in the previous five years were excluded from the present study (exclusion criteria). All the clinical procedures were approved by the Committees of Clinical Ethics of the Shenzhen People's Hospital and conformed to the principles of the Helsinki Declaration. We obtained informed consent from all the participants.

### Aged Mice

The C57BL/6 mice used in the present study were purchased from the Laboratory Animal Center of Air Force Medical University (Xi'an, Shaanxi, China). The mice were maintained in normal humidity (55-60%), temperature (25 °C), and light-dark (12 h/12 h) SPF conditions, given free access to water and feed. Naturally aged male mice used in this study were monitored continuously until the mice were more than 20-month-old, and the unhealthy mice were excluded. All mouse experiments were in accordance with the Guide for the Care and Use of Laboratory Animals and approved by the Laboratory Animal Ethics & Welfare Committee of Northwestern Polytechnical University.

### The osteoblastic miR-138-5p transgenic mouse model

The osteoblastic miR-138-5p transgenic (TG) mouse model was constructed as described previously 14. In brief, the recombinant plasmid including the miR-138-5p sequence with osteoblastic marker *Runx2* promoter was microinjected into a zygote to obtain stable TG mouse model. In this study, by mating the 2-6 month-old male TG mice with 2-4 month-old wild-type female mice, the genotype of transgenic mice were +/- mice, and the genotype of littermate WT mice were -/- mice.

### RNA extraction and Real-time PCR

High-quality total RNA was extracted from tissue or cell samples by the Omega miRNA kit (Omega, USA) according to the manufacturer's instruction. The quality of RNA was then tested by ultraviolet spectrophotometry. For miRNA and mRNA, both cDNAs were synthesized with the PrimeScript™ RT reagent Kit (Takara, Japan). Real-time PCR was performed by the using of SYBR^®^ Premix Ex Taq™ II kit (Takara, Japan) following the manufacturer's instruction. The mRNAs and miRNAs relative expression was analyzed by the 2^-ΔΔCT^ method. What's more, *Gapdh* was used as an internal control for mRNAs, and U6 was used as an internal control for miRNAs.

### Primary osteoblast isolation and cell culture

**Figure 1:**
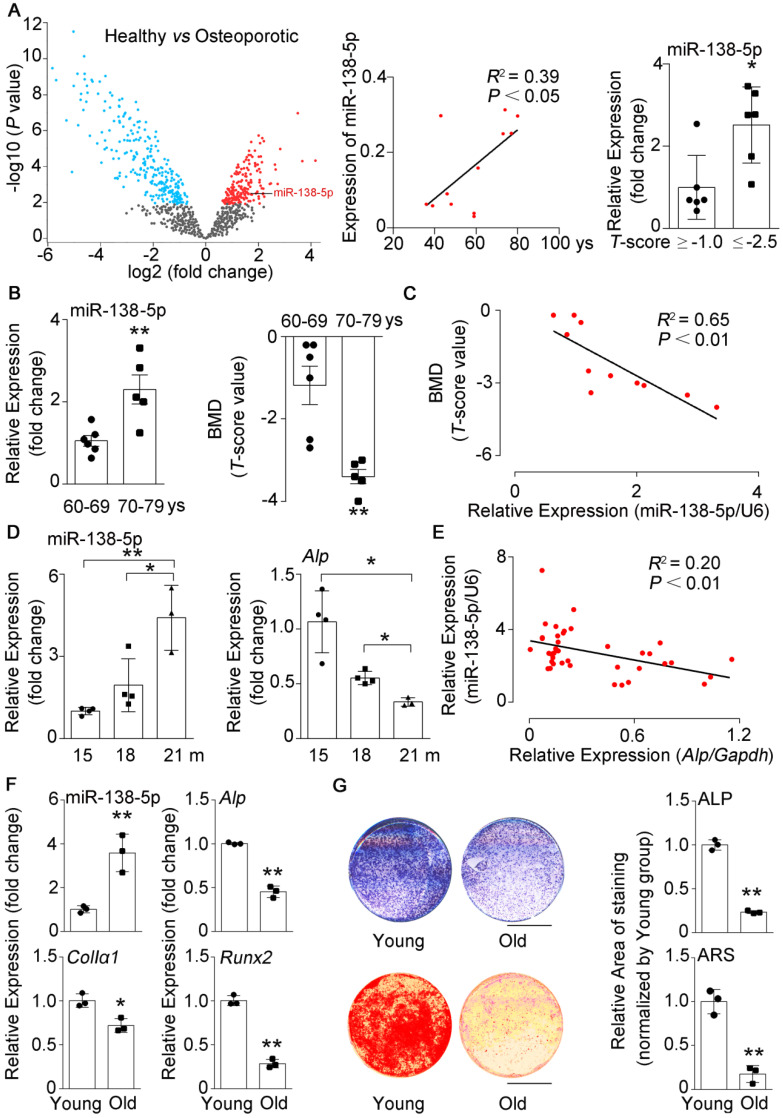
** miR-138-5p levels are elevated with age in humans, mice and cultured cells.** (**A**) The volcano plot analysis of differentially expressed miRNAs (left), linear regression analysis of miR-138-5p expression and age association (middle), relative miR-138-5p expression (right) in plasma of healthy patients (*T*-score ≥ -1, n = 6) or osteoporotic patients (*T*-score ≤ -2.5, n = 6). (**B**) Real-time PCR analysis of miR-138-5p (left) in bone specimens, and *T*-score (right) of spine from aged female fracture patients (60-69 years, n = 6; 70-79 years, n = 5). (**C**) Linear regression analysis of miR-138-5p and *T*-score association in aged female fracture patients. (**D**) Real-time PCR analysis of miR-138-5p (left) and *Alp* (right) levels in tibias from 15, 18 and 21-month-old male C57BL/6 mice, respectively. (15 m, n = 4; 18 m, n = 4; 21 m, n = 3). (**E**) Linear regression analysis of miR-138-5p and *Alp* association in wild-type C57BL/6 mice with different age. n = 39. (**F**) Real-time PCR analysis of miR-138-5p and osteogenic marker gene (*Alp*, *ColIa1* and *Runx2*) mRNA levels in primary osteoblasts from 6-month-old (young) and 21-month-old (old) mice, respectively. n = 3 for each group. (**G**) Representative images of ALP staining (upper, left) and quantification of ALP staining areas (upper, right), and Alizarin Red S (ARS) staining (lower, left, 10 d) and quantification of ARS staining areas (lower, right) in primary osteoblasts from 6-month-old (young) and 21-month-old (old) mice, respectively. Scale bar, 5 mm. n = 3 for each group. U6 small nuclear RNA was used as the internal control for miR-138-5p. *Gapdh* was used as the internal control for mRNAs. For human experiments, Data are represented as mean ± sem. For mice and cells experiments, Data are represented as mean ± s.d. Statistical differences among three groups were analyzed via one-way ANOVA and significances were determined using student's *t*-test between two groups. *P* value less than 0.05 was considered significant in all cases (*P < 0.05, **P < 0.01).

Primary osteoblasts in Figure 1 were isolated from the femora and tibias of 6-month-old or 21-month-old male mice according to bone research protocol 18. In brief, femora and tibias were excised with removing all muscles and placed in a petri dish with the α-MEM medium after euthanize adult mice. Then, the epiphyses of both ends were cut off and the bone marrow was flushed out by 27-gauge needle. The cleaned diaphyses were cut into little pieces of approximately 1-2 mm^2^ using scissors and washed with α-MEM medium several times. The bone pieces were incubated in collagenase II (Gibco, USA) solution at 37 °C in a shaking watch bath (150-200 rpm) for 2 hours (4 times, 30 min every time). From the fifth to the seventh incubation (30 min every time), primary long bone osteoblasts were collected for culture.

Mouse primary calvarial osteoblasts in Supplement Figure 4 were isolated from the calvaria of 1-2 d neonatal mice pups according to bone research protocol 18. Briefly, the calvaria of neonatal mice pups was excised with removing the edges and sutures and placed in a petri dish with the α-MEM medium. The calvaria was washed with α-MEM medium for several times. Then cleaned calvaria was incubated in collagenase I (Gibco, USA) solution at 37 °C in a shaking watch bath (150-200 rpm) for 2 hours and removed into a 6-well plate for culture.

The primary osteoblasts were maintained in α-MEM (Gibco, USA) with 10% FBS (Corning, USA), 1% penicillin and streptomycin (Amresco, USA). The primary osteoblasts were cultured under 5% CO_2_, 95% humidity and 37 °C incubator, and confluent cells were passaged with 0.25% trypsin. The primary osteoblasts were not used beyond passage 4.

### Construction of stable osteoblastic cell line and cell culture

For the construction of the stable miR-138-5p overexpression osteoblastic cell line (Hi-138) in murine osteoblastic MC3T3-E1 cells, recombinant lentivirus vectors carrying miR-138-5p precursor or one scrambled negative sequence were designed and synthesized by Genepharma (Shanghai, China) as described previously 15. For the construction of the stable knockdown MACF1 osteoblastic cell line (KD-Macf1) in MC3T3-E1 cells, recombinant lentivirus vectors carrying shRNA targeting mice Macf1 (NM_001199136.1) or its scramble control were designed and synthesized by Genepharma (Shanghai, China) as described previously 19. The cells were maintained in α-MEM supplemented with 10% FBS (Biological Industries, Israel), 1% penicillin and streptomycin (Amresco, USA). The cells were cultured under 5% CO_2_, 95% humidity and 37 °C incubator, and confluent cells were passaged with 0.25% trypsin.

### Cell transfection

The osteoblasts were seeded at plates with a density of 10^5^ cells/well until the confluence reached 80% ~ 90%, cells were transfected with 50 nM antagomir-138-5p or agomir-138-5p, or 1 μg/ml MACF1 siRNA by lipofectamine 2000 (invitrogen, USA) following the manufacturer's instructions. The serum-free medium was changed as the standard α-MEM medium after 6-8 h. The osteoblasts were incubated at 37 °C for 48 h and then harvested for real-time PCR, western blot analysis and cell staining. Antagomir-138-5p (Gene Pharma, China) sequences were 5'-CGGCCUGAUUCACAACACCAGCU-3'. Agomir-138-5p (Ribobio, China) sequences were 5'- AGCUGGUGUUGUGAAUCAGGCCG-3' (positive-sense strand), 5'-UCGACCACAACACUUAAUCCGGC-3' (antisense strand), MACF1 siRNA (Xi'an RQC, China) effective sequences were 5'-GCUAGUGAAUAUCCGCAAUGA-3'.

### Alkaline phosphatase (ALP) staining

Alkaline phosphatase staining was examined by BCIP/NBT alkaline phosphatase color development kit (Beyotime, China) as reported previously 14, 15, 20. Briefly, cells were fixed and rinsed with PBS. Then fixed cells were added BCIP/NBT liquid substrate until the color of cells turned blue/purple. Finally, cells were rinsed in deionized water for several times. The plate with stained cells was imaged with a scanner (Canon, Japan).

### Alizarin Red S (ARS) staining

For osteoblast differentiation, the osteoblasts were cultured in medium with 10% FBS, 1% streptomycin and penicillin, 50 μg/ml ascorbic acid (Sigma, USA) and 10 mM β-glycerophosphate (Sigma, USA) for about 2-3 weeks. Then, the osteoblasts were fixed and rinsed with PBS. The fixed cells were subsequently stained with 0.5% alizarin red s (Sigma, USA) solution (pH 4.2) for 15-30 min. Finally, cells were rinsed in deionized water for several times. The plate with stained cells was imaged with a scanner (Canon, Japan).

### Aged osteoblast induced by etoposide

The osteoblasts were seeded at plates at a density of 10^5^ cells/well. Cells were added with 2 μM etoposide (Solarbio, China) subsequently upon reaching 70% ~ 80% confluency. 48 h later, the medium was changed as the standard α-MEM medium. The osteoblasts were for cell transfection or incubated at 37 °C for 48 h and then harvested for real-time PCR, western blot analysis and cell staining.

### β-galactosidase staining

β-galactosidase activity was examined by β-galactosidase staining kit according to manufacturer's instructions (Beyotime, China). Briefly, cells were fixed and rinsed with PBS. Then fixed cells were added β-galactosidase liquid substrate for 24-48 h. Finally, cells were rinsed in deionized water for several times.

### Micro-computed tomography (micro-CT) analysis

The femur samples were fixed in 4% paraformaldehyde and scanned by micro-CT devices (μCT40, Switzerland) to assess and image bone microarchitecture in Figure 3 and Supplement Figure 5 as reported previously 7, 14. Briefly, the scanning resolution was set as 10 μm and 423 slices were scanned from the growth plate of the distal femur beginning. For micro-CT analysis, 80 continuous slices were selected as the region of interest (ROI) from beginning the distal growth plate disappearing to calculate the following parameters, bone mineral density (BMD), bone volume to tissue volume (BV/TV), trabecular spacing (Tb.Sp), trabecular number (Tb.N), trabecular thickness (Tb.Th) and structure model index (SMI). For 3D reconstruction of trabecular bone, the related parameters were set as sigma = 1.2, supports = 2 and threshold = 200.

The femur samples were fixed in 4% paraformaldehyde and scanned by micro-CT devices (General Electric, WI) to assess and image bone microarchitecture in Figure 6 and Supplement Figure 1 as reported previously 14, 21. Briefly, the scanning parameters were set as following, Energy: 80 kVp/80 μA; Angle of Increment: 0.5°; Exposure Time: 3000 ms/frame; Scanning Time: 120 min and the scanning resolution were set as 8 μm. For micro-CT analysis, 1 mm was selected as the region of interest (ROI) from beginning the distal growth plate disappearing to calculate the following parameters, bone mineral content (BMC), BMD, BV/TV, Tb.N, Tb.Th and Tb.Sp. The 3D reconstruction of trabecular bone was used by the microview software.

### Bone mechanical property

The three-point bending mechanical test system (UniVert, Canada) was used to examine the bone mechanical properties of tibias. The anterior surface on the two lower support points spaced 10 mm apart. The tibias were fixed on a bracket with a loading speed of 0.6 mm/min until fracture occurred at the anterior surface on the two lower support points spaced 10 mm apart. Load-displacement curves were acquired and used to calculate the maximum load at failure, stiffness and elasticity modulus.

### Double calcein labeling

All mice were intraperitoneally injected with 10 mg/kg calcein green (Sigma, USA) at 10 d and 2 d before euthanasia. After dehydration and embedding into gradient methyl methacrylate (MMA), the fixed femur samples were then sliced into 20-40 μm sections by the EXAKT 300CP tissue cutting system and the EXAKT 400CS micro grinding system (EXAKT Apparatebau, Germany). Sections were examined with a fluorescence microscope (Nikon, Japan), bone dynamic parameter (MAR) was calculated with ImageJ software (NIH, USA) 14, 20.

### Bone histological decalcified sections staining

The femurs were fixed with 4% PFA for 2 days, decalcified with EDTA for two weeks, and then embedded in paraffin. Serial 4 μm thick tissue sections for hematoxylin and eosin (HE) staining, Masson's trichrome staining or immunohistochemical (IHC) staining following the manufacturer's instructions as we have reported (Servicebio, China) 14, 22. The stained sections were scanned by a Digital Whole Slide Scanner (Leica, Germany).

### Western blot

The osteoblasts were harvested and then lysed with Cell Lysis Buffer (Beyotime, China) containing a protease inhibitor cocktail (Merck, Germany) on ice. The protein extracts were collected after 4 °C centrifugation (15,000 g for 5 min) and then boiled with SDS-PAGE protein loading buffer (Biosharp, China). The protein was transferred to nitrocellulose membranes (PALL, USA) after separation via 8% SDS-PAGE (MACF1) or 10% SDS-PAGE (COLIα1 and GAPDH). The separated protein fractions on the membranes were blocked with 5% skim milk and incubated overnight at 4 °C with specific primary antibodies on a shaking device. HRP-labeled secondary antibody (Beijing CoWin Bioscience, China) was added after primary antibodies rinsed off, and then protein bands were visualized using ECL chemiluminescence reagents (Rockford, United States) by X-ray film (Kodak, NY) or T5200 Multi Chemiluminescence Detection System (Tanon, China). The primary antibodies included MACF1 Rabbit pAb (1:1000, Abcam, USA), COLIα1 Rabbit pAb (1:1000, Cell Signaling Technology, USA) and GAPDH Rabbit pAb (1:1000, Calbiochem, Germany) 23.

### Dual-luciferase reporter assay

MACF1 3′UTR vector and its mutant vector which we previously reported 14 were used to further detect direct interaction between MACF1 and miR-138-5p. Briefly, the reporter plasmids were co-transfected with pRL-TK Renilla plasmid into MC3T3-E1 cells (8×10^6^ cells per well), and antagomir-138-5p or antagomir-NC transfection was performed as reported 14. The osteoblasts were harvested for the luciferase assay 48 h after transfection, and then performed with the dual-luciferase reporter assay system (Promega, USA) according to the manufacturer's instructions. Each value of quantified luminescent signals from the firefly luciferase was normalized by Renilla luciferase.

### Antagomir-138-5p therapy in aged mice

20-month-old male C57BL/6 mice received antagomir-138-5p (antagomir) or antagomir-NC (10 mg/kg body weight) with the osteoblast-targeted delivery system 7 by tail vein injection for four consecutive injections every two weeks. The groups were as followed: 22 m group (no treatment), 22 m + Veh group (injection of the osteoblast-targeted delivery system alone), 22 m + antagomir-NC group (injection of antagomir-NC with the osteoblast-targeted delivery system) and 22 m + antagomir group (injection of antagomir-138-5p with the osteoblast-targeted delivery system). Before euthanasia, all mice were intraperitoneally injected with 10 mg/kg calcein green at 10 d and 2 d before euthanasia.

### Statistical analysis

For data consisting of two independent variables, two-way analysis of variance (ANOVA) was performed to study the interaction between fixed factors. Then, statistical differences among three or more groups were analyzed via one-way ANOVA and significance between two groups was determined using Student's *t*-test. For human experiments, all numerical data were presented as the mean ± sem. For the mouse and cell experiments, all numerical data were presented as the mean ± sd. All statistical analyses were performed with GraphPad Prism 6 software and *P* < 0.05 was considered statistically significant for all comparisons (*P < 0.05, **P < 0.01).

## Results

### miR-138-5p levels are elevated with age in humans, mice and cells

In our previous study, we found that silencing of mechanoresponsive miR-138-5p could partially neutralize disuse osteoporosis 14. Interestingly, our miRNA expression profile (GSE93883) analysis of plasma of healthy or osteoporotic patients showed that the miR-138-5p expression was increased with age and elevated in osteoporotic patients (*T*-score ≤ -2.5) (**Figure 1A**). Hence, the role of miR-138-5p in senile bone loss attracted our attention. Furthermore, we have previously confirmed that the miR-138-5p level was increased with age in bone specimens of aged male osteoporotic patients 14. Here, we further detected and verified that the expression of miR-138-5p was also elevated and spine bone mineral density (*T*-score) was significantly decreased in bone specimens of female osteoporotic patients (**Figure 1B**). Moreover, the miR-138-5p level was negatively correlated to *T*-score (**Figure 1C**). Furthermore, the relationship between miR-138-5p level and age was validated in bone tissue of mice and primary osteoblasts, and the results showed that the expression of miR-138-5p was increased with age at mice or cellular levels, respectively (**Figure 1D, F**). In contrast, the bone architecture, trabecular bone mass-related parameters (bone mineral density (BMD), bone volume to tissue volume (BV/TV), trabecular thickness (Tb.Th.), trabecular t (Tb.N.)) and markers of osteoblast differentiation or bone formation (osteogenic marker genes expression, alkaline phosphatase (ALP) activity and matrix mineralization) were inversely correlated with miR-138-5p level in bone tissue and primary osteoblasts of aged mice (**Figure 1D-G, Figure S1A, B**). These results suggested that miR-138-5p is an aging-related miRNA and that its level was negatively correlated to osteoblast differentiation and bone formation in human tissues, mice and cultured cells.

### High miR-138-5p levels aggravate the decrease in osteoblast differentiation induced by aging

**Figure 2:**
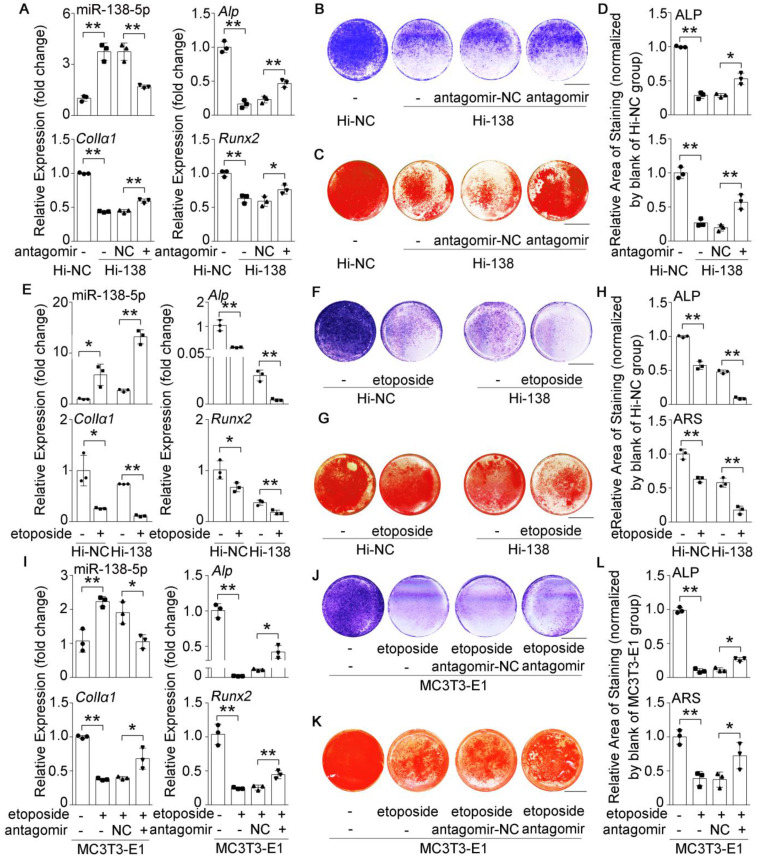
** High miR-138-5p levels aggravate the decrease in osteoblast differentiation induced by aging.** (**A**) Real-time PCR analysis of miR-138-5p and osteogenic marker gene (*Alp*, *ColIa1* and* Runx2*) mRNA levels in stable miR-138-5p overexpression osteoblastic cell line (Hi-138) treating with either antagomir-138-5p (antagomir) or antagomir-NC (NC), or in negative control cell line (Hi-NC) for 48 h, respectively. n = 3 for each group. (**B**) Representative images of ALP staining in Hi-138 cells treating with either antagomir-138-5p or antagomir-NC, or in Hi-NC cells for 48 h, respectively. Scale bar, 5 mm. (**C**) Representative images of ARS staining in Hi-138 cells treating with either antagomir-138-5p or antagomir-NC, or in Hi-NC cells for 14 d respectively. Scale bar, 5 mm. (**D**) Quantification of ALP staining areas (upper) and ARS staining areas (lower, 14 d) in Hi-138 cells treating with either antagomir-138-5p or antagomir-NC, or in Hi-NC cells, respectively. n = 3 for each group. (**E**) Real-time PCR analysis of miR-138-5p and osteogenic marker gene (*Alp*, *ColIa1* and* Runx2*) mRNA levels in Hi-138 cells or Hi-NC cells treating with 2-day etoposide-induced aging, respectively. n = 3 for each group. (**F**) Representative images of ALP staining in Hi-138 cells or Hi-NC cells treating with 2-day etoposide inducing aging, respectively. Scale bar, 5 mm. (**G**) Representative images of ARS (18 d) staining in Hi-138 cells or Hi-NC cells treating with 2-day etoposide-induced aging, respectively. Scale bar, 5 mm. (**H**) Quantification of ALP staining areas (upper) and ARS staining areas (lower, 18 d) in Hi-138 cells or Hi-NC cells treating with 2-day etoposide-induced aging, respectively. n = 3 for each group. (**I**) Real-time PCR analysis of miR-138-5p and osteogenic marker gene (*Alp*, *ColIa1* and* Runx2*) mRNA levels in MC3T3-E1 cells treating with either antagomir-138-5p or antagomir-NC after 2-day etoposide-induced aging for 48 h, respectively. n = 3 for each group. (**J**) Representative images of ALP staining in MC3T3-E1 cells treating with either antagomir-138-5p or antagomir-NC after 2-day etoposide-induced aging for 48 h, respectively. Scale bar, 5 mm. (**K**) Representative images of ARS staining (14 d) in MC3T3-E1 cells treating with either antagomir-138-5p or antagomir-NC after 2-day etoposide-induced aging, respectively. Scale bar, 5 mm. (**L**) Quantification of ALP staining areas (upper) and ARS staining areas (lower, 14 d) in MC3T3-E1 cells treating with either antagomir-138-5p or antagomir-NC after 2-day etoposide-induced aging, respectively. n = 3 for each group. U6 small nuclear RNA was used as the internal control for miR-138-5p, and *Gapdh* was used as the internal control for mRNAs. Data are represented as mean ± s.d. Two-way ANOVA was performed to study the interaction between two independent variables. Then, statistical differences among three or more groups were analyzed via one-way ANOVA and significances were determined using student's *t*-test between two groups. *P* value less than 0.05 was considered significant in all cases (*P < 0.05, **P < 0.01).

To further validate the roles of miR-138-5p in regulating osteoblast differentiation, we first detected the expression of miR-138-5p at different time points (1 day, 7 days, 14 days) in the osteogenic differentiation process. We found that the expression of osteogenic marker factors (*Alp*, *ColIα1*, *Runx2*) increased while miR-138-5p expression was gradually decreased during primary calvaria osteoblast differentiation (**Figure S2A, B**). Furthermore, primary calvaria osteoblasts were transfected with agomir-138-5p (miR-138-5p agonist, agomir) or antagomir-138-5p (miR-138-5p inhibitor, antagomir). Real-time PCR analysis confirmed that the expression level of intracellular miR-138-5p was significantly upregulated by agomir-138-5p, and substantially downregulated by antagomir-138-5p compared to their corresponding controls (**Figure S2C, G**). Functionally, the expression levels of *Alp*, *ColIα1*, and *Runx2*, ALP activity and matrix mineralization nodules formation were weakened by agomir-138-5p, whereas enhanced by antagomir-138-5p (**S-Figure 2C-J**). Previously, we have constructed a stable miR-138-5p overexpression osteoblastic cell line (Hi-138) in murine osteoblastic MC3T3-E1 cells with lentivirus 15. Compared to the negative control cell line (Hi-NC), Hi-138 cells showed higher miR-138-5p level and lower levels of osteoblast differentiation markers (marker gene expression (Alp, ColIα1, and Runx2), ALP activity and matrix mineralization nodule formation)). However, silencing of miR-138-5p could partly rescue the reduction of osteoblast differentiation markers induced by high miR-138-5p level in Hi-138 cells treating with antagomir-138-5p (**Figure 2A-D**).

To further identify the effects of miR-138-5p on aging-related decreased osteoblast differentiation, we used etoposide to induce senescence in Hi-138 cells or Hi-NC cells. The results showed that the miR-138-5p level and cell senescence were increased, while osteoblast differentiation markers were decreased after treatment with etoposide. Moreover, the decrease in osteoblast differentiation induced by elevated miR-138-5p level was aggravated by etoposide-induced aging (**Figure 2E-H, Figure S3A, C, D**). In addition, the decreases in *Alp*, *ColIα1*, and *Runx2* mRNA levels, COLIα1 protein level, ALP activity and matrix mineralization nodules formation, and the increases in cell senescence induced by etoposide-induced aging were partially neutralized by transfection with antagomir-138-5p in osteoblastic MC3T3-E1 cells (**Figure 2I-L, Figure S3B, E, F**). These results together demonstrated that high miR-138-5p level could exacerbate aging-related reduced osteoblast differentiation *in vitro*.

### High miR-138-5p levels aggravate aging-related bone loss

**Figure 3:**
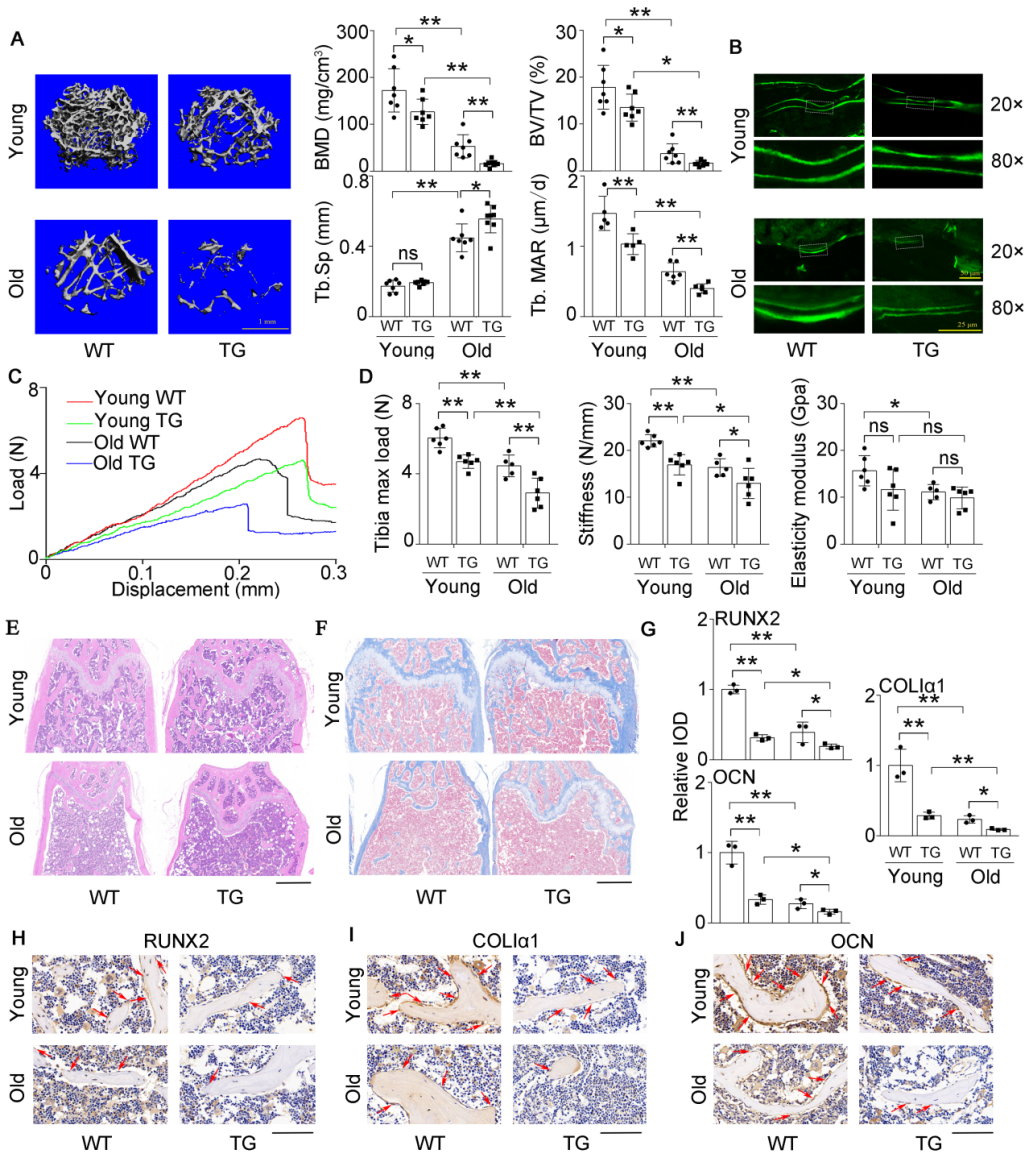
** High miR-138-5p levels aggravate aging-related bone loss.** (**A**) Representative 3D reconstruction images showing microarchitecture, and microCT analysis of BMD, BV/TV and Tb.Sp in distal femur of young or old osteoblastic miR-138-5p transgenic (TG) mice and wildtype (WT) mice, respectively. BMD, bone mineral density; BV/TV, Bone volume per tissue volume; Tb.Sp, trabecular spacing, young, 3-month-old, old, 19-month-old. Scale bar, 1 mm. Young-WT, n = 7; Young-TG, n = 7; Old-WT, n = 7; Old-TG, n = 8. Dynamic histomorphometric analysis of MAR showing bone formation capacity in femur trabecular (lower, right) bone of young or old TG mice and WT mice, respectively. MAR, mineral apposition rate. Young-WT, n = 5; Young-TG, n = 5; Old-WT, n = 6; Old-TG, n = 5. (**B**) Representative calcein double labeling images showing bone formation capacity in femur trabecular bone of young or old TG mice and WT mice. Upper scale bar, 50 µm. Lower scale bar, 25 µm. young, 3-month-old, old, 19-month-old. (**C**) Representative load-deflection curves for the respective groups in tibias of young or old TG mice and WT mice. young, 3-month-old, old, 19-month-old. (**D**) Three-point bending measurement of tibia max load, stiffness and elasticity modulus in young or old TG mice and WT mice. young, 3-month-old, old, 19-month-old. Young-WT, n = 6; Young-TG, n = 6; Old-WT, n = 5; Old-TG, n = 6. (**E**) Representative images of H&E staining analysis in distal femur of young or old TG mice and WT mice. Scale bar, 500 µm. young, 3-month-old, old, 19-month-old. (**F**) Representative images of Masson's trichrome staining analysis in distal femur of young or old TG mice and WT mice. Scale bar, 500 µm. young, 3-month-old, old, 19-month-old. (**G, H**) Representative images of immunohistochemical (IHC) staining and quantification analysis of RUNX2 (left), COLIα1 (middle) and OCN (right) in distal femur of young or old TG mice and WT mice. Scale bar, 100 µm. young, 3-month-old, old, 19-month-old. n = 3 mice for each group. Data are represented as mean ± s.d. Two-way ANOVA was performed to study the interaction between two independent variables. Significances were determined using student's *t*-test between two groups. *P* value less than 0.05 was considered significant in all cases (*P < 0.05, **P < 0.01).

Previously, we constructed an osteoblastic miR-138-5p transgenic (TG) mouse model by the *Runx2* promoter 14 and found that the bone formation activity of TG mice was decreased compared to that of wild-type (WT) mice. Here, the expression of *Alp*, *ColIα1*, and *Runx2*, ALP activity and matrix mineralization were significantly suppressed in primary calvarial osteoblasts isolated from TG mice, compared to WT mice (**Figure S4A-D**). To further investigate the effect of miR-138-5p on aging-related bone loss *in vivo*, TG mice and WT mice were divided into young (3-month) and old (19-month) groups. We scrutinized bone mass and trabecular microarchitecture and found that the bone mineral density and bone mass in the TG mice declined significantly compared to that in the controls in both the young group and old group, and the trabecular microarchitecture appeared to have deteriorated in TG mice. These phenomena worsened with age (**Figure 3A, Figure S5A**). The calcein-labeled bone formation experiment showed that the bone formation rate and osteoblast number decreased in TG mice and declined more with age (**Figure 3A, B and Figure S5B**). With increasing age, the skeletal mechanical strength properties (Tibia max load and stiffness) of mice tibias in the TG groups were significantly weaker compared to those in the WT groups (**Figure 3C, D**). HE staining and Masson's trichrome staining images showed that TG mice had loose bone trabeculae compared to WT mice, this phenotype was more severe in older mice (**Figure 3E, F**). As expected, the IHC assay also showed that the expression levels of osteogenic genes (Runx2, ColIα1and Ocn) were lower in TG mice than in WT mice. Likewise, the expression levels of these genes further decreased with age (**Figure 3G-J**). These results indicated that high miR-138-5p level could exacerbate aging-related bone loss and the decreased in bone formation *in vivo*.

### miR-138-5p suppresses osteoblast differentiation by targeting and inhibiting MACF1 in senile bone loss

**Figure 4:**
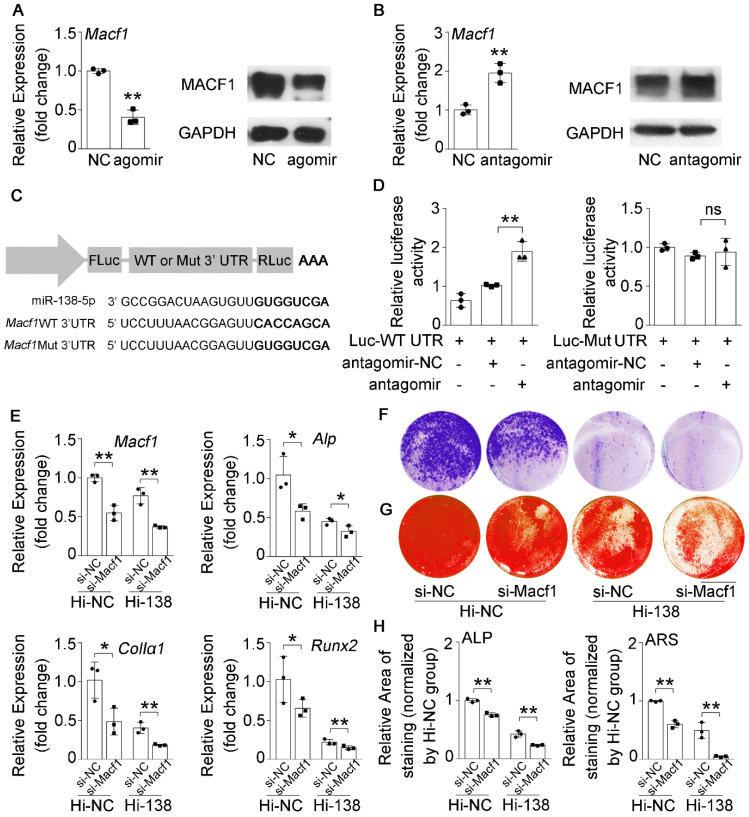
** miR-138-5p suppresses osteoblast differentiationby targeting and inhibiting MACF1.** (**A**) Real-time PCR (left) and Western blot (right) analysis of MACF1 expression level in MC3T3-E1 cells treating with either agomir-138-5p (agomir) or agomir-NC for 48 h, respectively. n = 3 for each group. (**B**) Real-time PCR (left) and Western blot (right) analysis of MACF1 expression level in MC3T3-E1 cells treating with either antagomir-138-5p (antagomir) or antagomir-NC for 48 h, respectively. n = 3 for each group. (**C**) Schematic graph showing the design of dual-luciferase reporters with either *Macf1* WT 3'UTR or *Macf1* mutant 3'UTR. (**D**) The effects of antagomir-138-5p (antagomir) or antagomir-NC on luciferase activity of *Macf1* WT 3'UTR reporter (Luc-WT UTR, left), Macf1 mutant 3'UTR reporter (Luc-Mut UTR, right) in MC3T3-E1 cells, respectively (left). (**E**) Real-time PCR analysis of *Macf1* and osteogenic marker gene (*Alp*, *ColIa1* and* Runx2*) mRNA levels in stable miR-138-5p overexpression osteoblastic cell line (Hi-138) treating with either MACF1 siRNA (si-Macf1) or negative control siRNA (si-NC), or in negative control cell line (Hi-NC) for 48 h, respectively. n = 3 for each group. (**F**) Representative images of ALP staining in Hi-138 cells or Hi-NC cells treating with either si-Macf1 or si-NC for 48 h, respectively. Scale bar, 5 mm. (**G**) Representative images of ARS (18 d) staining in Hi-138 cells or Hi-NC cells treating with either si-Macf1 or si-NC for 48 h, respectively. Scale bar, 5 mm. (**H**) Quantification of ALP staining areas (upper) and ARS staining areas (lower, 18 d) in Hi-138 cells or Hi-NC cells treating with either si-Macf1 or si-NC for 48 h, respectively. n = 3 for each group. *Gapdh* was used as the internal control for mRNAs. Data are represented as mean ± s.d. Two-way ANOVA was performed to study the interaction between two independent variables. Then, statistical differences among three or more groups were analyzed via one-way ANOVA and significances were determined using student's *t*-test between two groups. *P* value less than 0.05 was considered significant in all cases (*P < 0.05, **P < 0.01).

MiR-138-5p has been demonstrated to directly target MACF1 to regulate osteoblast differentiation in our previous study 14. However, the relationship between miR-138-5p and MACF1 in senile bone loss needs further validation. We determined that MACF1 expression was inhibited or promoted after transfecting MC3T3-E1 osteoblasts with agomir-138-5p or antagomir-138-5p, respectively (**Figure 4A, B and Figure S6A, B**). We then cloned the binding region or the mutant sequence into a dual luciferase reporter vector (**Figure 4C**). We found that luciferase activity was significantly elevated in the cells simultaneously transfected with antagomir-138-5p and *Macf1* WT 3' UTR plasmid, while plasmids bearing mutations inmiR-138-5p or the *Macf1* 3' UTR could not change the luciferase activity significantly (**Figure 4D**). Additionally, we used Macf1-siRNA (si-Macf1) to knock down Macf1 in miR-138-5p high expression (Hi-138) cells. The results showed that the expressions of *Macf1* and osteogenic genes (*Alp*, *ColIα1*, and *Runx2*) mRNA levels, ALP activity and matrix mineralization nodules formation were further attenuated after MACF1 deficiency in Hi-138 cells, compared to Hi-NC cells (**Figure 4E-H**). These data suggested that miR-138-5p directly targeted MACF1 to regulate osteoblast differentiation.

**Figure 5:**
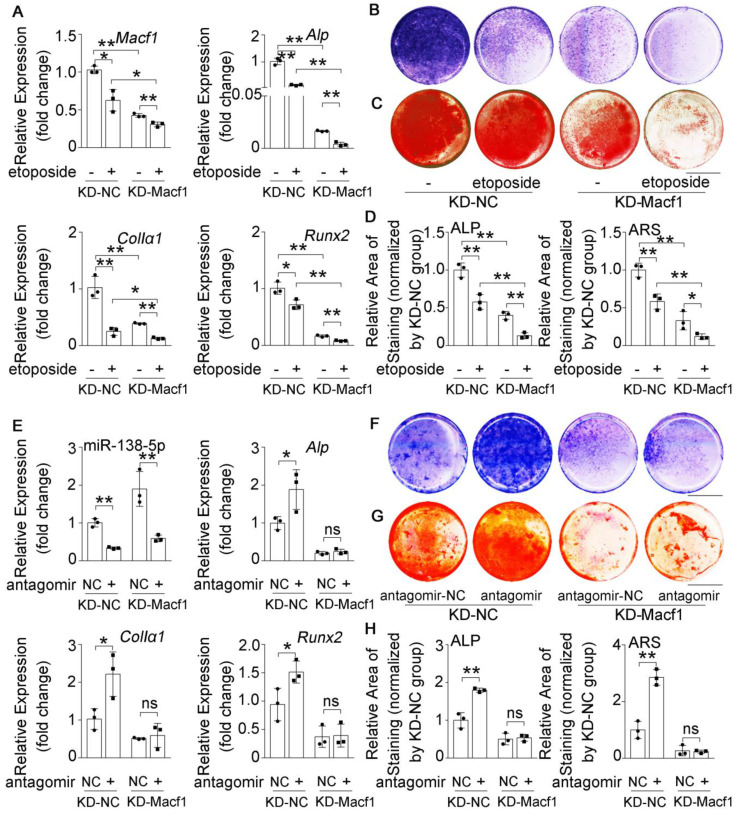
** MACF1 is involved in miR-138-5p regulating aged osteoblast differentiation.** (**A**) Real-time PCR analysis of *Macf1* and osteogenic marker gene (*Alp*, *ColIa1* and* Runx2*) mRNA levels in stable MACF1 low-expressing (KD-Macf1) cells treating with 2-day etoposide-induced aging, or in negative control cell line (KD-NC) for 48 h, respectively. n = 3 for each group. (**B**) Representative images of ALP staining in KD-Macf1 cells or KD-NC cells either treating with 2-day etoposide-induced aging for 48 h, respectively. Scale bar, 5 mm. (**C**) Representative images of ARS (16 d) staining in KD-Macf1 cells or KD-NC cells treating with 2-day etoposide-induced aging for 48 h, respectively. Scale bar, 5 mm. (**D**) Quantification of ALP staining areas (upper) and ARS staining areas (lower, 16 d) in KD-Macf1 cells or KD-NC cells treating with 2-day etoposide-induced aging for 48 h, respectively. n = 3 for each group. (**E**) Real-time PCR analysis of miR-138-5p and osteogenic marker gene (*Alp*, *ColIa1* and* Runx2*) mRNA levels in KD-Macf1 cells or KD-NC cells treating with either antagomir-138-5p or antagomir-NC for 48 h, respectively. n = 3 for each group. (**F**) Representative images of ALP staining in KD-Macf1 cells or KD-NC cells treating with either antagomir-138-5p or antagomir-NC for 8 d, respectively. Scale bar, 5 mm. (**G**) Representative images of ARS staining (14 d) in KD-Macf1 cells or KD-NC cells treating with either antagomir-138-5p or antagomir-NC for 48 h, respectively. Scale bar, 5 mm. (**H**) Quantification of ALP staining areas (upper) and ARS staining areas (lower, 14 d) in KD-Macf1 cells or KD-NC cells treating with either antagomir-138-5p or antagomir-NC for 48 h, respectively. n = 3 for each group. U6 small nuclear RNA was used as the internal control for miR-138-5p. *Gapdh* was used as the internal control for mRNAs. Data are represented as mean ± s.d. Two-way ANOVA was performed to study the interaction between two independent variables. Significances were determined using student's *t*-test between two groups. *P* value less than 0.05 was considered significant in all cases (*P < 0.05, **P < 0.01).

To further validate the relationship between miR-138-5p and MACF1 during aging-related bone loss, we first treated the stable MACF1 low-expressing (KD-*Macf1*) cells with etoposide to induce osteoblast senescence (**Figure S6E, F**). The results showed that *Macf1* and osteogenic gene (*Alp*, *ColIα1*, and *Runx2*) mRNA levels, COLIα1 protein level, ALP activity and matrix mineralization were further attenuated after etoposide-induced aging in MACF1-deficient osteoblasts (**Figure 5A-D, Figure S6C-E**), which was similar to the previously observed osteoblast differentiation phenomenon in which miR-138-5p high-expressing osteoblasts were treated with etoposide to induce aging or si-*Macf1* to induce *Macf1* deficiency. Furthermore, we transfected antagomir-138-5p into either KD-*Macf1* cells or negative control (KD-NC) cells. In KD-NC osteoblasts, the miR-138-5p level was significantly decreased and osteogenic marker gene expression, COLIα1 protein level, ALP activity and the formation of mineralized nodules were increased after treating with antagomir-138-5p. However, decreasing the miR-138-5p level in KD-Macf1 osteoblasts led to a no significant difference in the markers of osteoblast differentiation (**Figure 5E-H, Figure S6F**). These results suggested that in aging-related reduced osteoblast differentiation, miR-138-5p regulated osteogenic differentiation by targeting and inhibiting MACF1.

### Inhibition of miR-138-5p can alleviate aging-related bone loss

**Figure 6:**
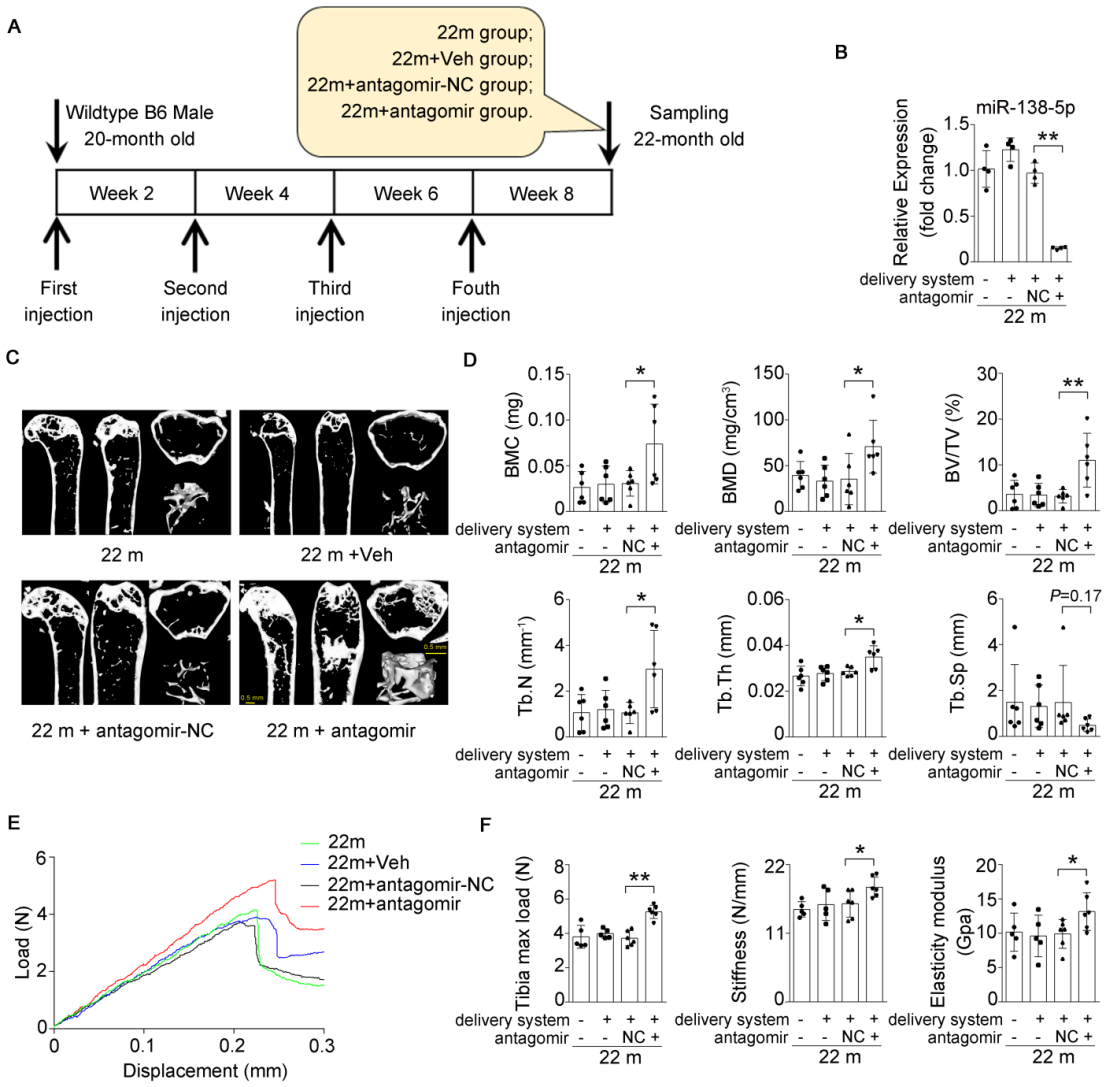
** Bone-targeted delivery of miR-138-5p inhibitor alleviates aging-related bone loss.** (**A**) Schematic graph showing experiment grouping, antagomir-138-5p treatment in aged mice. Mice were injected with antagomir-138-5p four times every two weeks during two-month therapy. 22 m (22-month-old mice, without any injection), 22 m + Veh (22-month-old mice injected with osteoblast-targeted delivery system), 22 m + antagomir-NC (22-month-old mice injected with osteoblast-targeted delivery system and antagomir-NC), 22 m + antagomir (22-month-old mice injected with osteoblast-targeted delivery system and antagomir-138-5p). (**B**) Real-time PCR analysis of miR-138-5p relative level in bone tissue of aged mice after antagomir-138-5p treatment. n = 4 for each group. (**C**) Representative 3D reconstruction images showing microarchitecture in distal femur of aged mice after antagomir-138-5p treatment. Scale bar, 0.5 mm. (**D**) The microCT statistical analysis of BMC, BMD, BV/TV, Tb.Th, Tb.N and Tb.Sp in distal femur of aged mice after AMO treatment. BMC, bone mineral content; BMD, bone mineral density; BV/TV, bone volume to tissue volume; Tb.N, trabecular number; Tb.Th, trabecular thickness; Tb.Sp, trabecular spacing. n = 6 mice for each group. (**E**) Representative load-deflection curves for the respective groups in tibias of aged mice after antagomir-138-5p treatment. (**F**) Three-point bending measurement of tibia max load, stiffness and elasticity modulus in tibias of aged mice after antagomir-138-5p treatment. 22 m, n = 5; 22 m + Veh, n = 5; 22 m + NC, n = 6; 22 m + antagomir, n = 6. U6 small nuclear RNA was used as the internal control for miR-138-5p. Data are represented as mean ± s.d. Two-way ANOVA was performed to study the interaction between two independent variables. Then, statistical differences among three or more groups were analyzed via one-way ANOVA and significances were determined using student's *t*-test between two groups. *P* value less than 0.05 was considered significant in all cases (*P < 0.05, **P < 0.01).

Based on our findings that an increased miR-138-5p level was accompanied by bone loss with age and that silencing of miR-138-5p could partially decrease osteoblast differentiation induced by aging *in vitro* (Figure 1-3) 14, we hypothesized that therapeutic inhibition of miR-138-5p could rescue the aging-related bone loss *in vivo* as well. To test our hypothesis, we separately treated 20-month-old male wild-type C57/BL6 mice with pharmacological antagomir-138-5p (antagomir) or antagomir-NC (NC) carried by an osteoblast-targeting delivery system 7 for 4 consecutive injections via the tail vein every two weeks (**Figure 6A**). We first confirmed that antagomir-138-5p could effectively downregulate miR-138-5p level in aged bone tissue (**Figure 6B**). In addition, we performed microCT analysis and found that antagomir-138-5p relieved the deterioration of trabecular bone mass and trabecular microarchitecture caused by aging (**Figure 6C**). Furthermore, the trabecular bone parameter analysis showed that BMC, BMD, BV/TV, Tb.N and Tb.Th. were significantly rescued after treatment with pharmacological antagomir-138-5p (**Figure 6D**). To further investigate the functions of pharmacological antagomir-138-5p on tibial mechanical strength properties in aged mice, we performed a three-point bending mechanical test and found that the tibial max load, stiffness, and elasticity modulus were also significantly increased in the antagomir group (**Figure 6E, F**).

### Inhibition of miR-138-5p can alleviate the decrease in bone formation induced by aging

**Figure 7:**
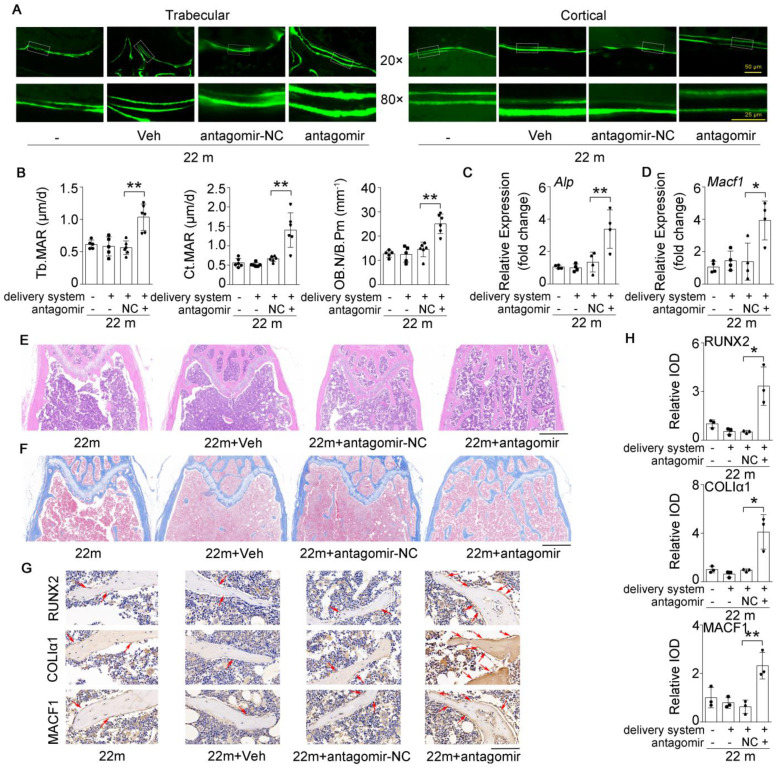
** Bone-targeted inhibition of miR-138-5p can alleviate the decrease in bone formation induced by aging.** (**A**) Representative calcein double labeling images showing bone formation capacity in femur trabecular and cortical bone of aged mice after antagomir-138-5p treatment. Upper scale bar, 50 µm. Lower scale bar, 25 µm. 22 m (22-month-old mice, without any injection), 22 m + Veh (22-month-old mice injected with osteoblast-targeted delivery system), 22 m + antagomir-NC (22-month-old mice injected with osteoblast-targeted delivery system and antagomir-NC), 22 m + antagomir (22-month-old mice injected with osteoblast-targeted delivery system and antagomir-138-5p). (**B**) Dynamic histomorphometric analysis of MAR showing bone formation capacity in femur trabecular (left) and cortical (middle) bone of aged mice after antagomir-138-5p treatment; Static histomorphometric analysis of OB.N/B.Pm (right) showing osteoblast number in femur trabecular bone of aged mice after antagomir-138-5p treatment. OB.N/B.Pm: osteoblast number per bone perimeter. 22 m, n = 5; 22 m + Veh, n = 5; 22 m + antagomir-NC, n = 6; 22 m + antagomir, n = 6. (**C, D**) Real-time PCR analysis of *Alp* and *Macf1* relative levels in bone tissue of aged mice after antagomir-138-5p treatment. n = 4 for each group. (**E**) Representative images of H&E staining analysis in distal femur of aged mice after antagomir-138-5p treatment. Scale bar, 500 µm. (**F**) Representative images of Masson's trichrome staining analysis in distal femur of aged mice after antagomir-138-5p treatment. Scale bar, 500 µm. (**G, H**) Representative images of immunohistochemical (IHC) staining and quantification analysis of RUNX2 (upper), COLIα1 (middle) and MACF1 (lower) in distal femur of aged mice after antagomir-138-5p treatment. Scale bar, 100 µm. n = 3 for each group. *Gapdh* was used as the internal control for mRNAs. Data are represented as mean ± s.d. Two-way ANOVA was performed to study the interaction between two independent variables. Then, statistical differences among three or more groups were analyzed via one-way ANOVA and significances were determined using student's *t*-test between two groups. *P* value less than 0.05 was considered significant in all cases (*P < 0.05, **P < 0.01).

Given that the osteoblastic miR-138-5p level was elevated and negatively correlated with bone formation in aged mice (**Figure 1 and Figure 3**), we further tested whether therapeutic inhibition of osteoblastic miR-138-5p could prevent the aging-related decrease in bone formation. Calcein double-labeling confirmed that aged mice treated with pharmacological antagomir-138-5p showed a significantly higher bone formation rate and related parameter (MAR) of trabecular and cortical bone than other normal control groups (**Figure 7A, B**). Moreover, HE staining and Masson's trichrome staining images showed that elevated trabecular bone and increased osteoid were observed in aged mice after pharmacological antagomir-138-5p treatment (**Figure 7E, F**). To further examine the expression of bone formation marker gene (Alp, Runx2 and ColIα1) and the miR-138-5p target MACF1, we performed the real-time PCR and IHC assay analysis. The results showed that the expression levels of osteogenic genes and MACF1 in the bone tissue of aged mice were significantly increased in the antagomir-138-5p group (**Figure 7C, D and Figure 7G, H**). These data suggested that the pharmacological miR-138-5p inhibitor could alleviate the decrease in bone formation and improve senile bone loss symptoms in aged mice.

## Discussion

In the present study, we identified miR-138-5p as a novel aging-related miRNA that negatively regulates osteogenesis by targeting and inhibiting MACF1 in aging-related bone loss. Especially for senile osteoporosis, elevated miR-138-5p levels led to greater decrease in osteoblast differentiation and more severe bone loss with aging. In addition, administering a miR-138-5p inhibitor to aged mice alleviated senile bone loss symptoms, which provided a new mechanism and a potential therapeutic strategy for the treatment of senile osteoporosis.

Senile osteoporosis is a growing public health problem worldwide, and it is essential to develop more effective approaches for its treatment 1, 2. It has been reported that osteoblast dysfunction inducing decreased bone formation may be one of the major causes of senile osteoporosis 9, 14, 24-29. In the current study, we built upon our previous study 14 and found that BMD and osteogenic marker gene levels gradually decreased with age in bone specimens from aged osteoporotic patients. Furthermore, bone formation and osteoblast differentiation were also reduced with aging in mice and cultured cells. Therefore, it is necessary to systematically clarify the mechanism by which aged osteoblast dysfunction leads to a decrease in osteogenesis during senile osteoporosis.

With the growing evidence of the important regulatory roles of miRNAs in aged osteoblast differentiation and aging-related bone loss 7-9, 25, 30-34, the relationship between miRNAs and senile osteoporosis has become a focus of substantial research. Recently, miR-146a was found to be an essential epigenetic switch controlling osteoblast generation and aging-related bone loss by regulating bone anabolic Wnt signaling 25. In addition, Sun et al. reported that miR-103-3p was negatively correlated with bone formation in bone specimens from elderly women with fractures 9. Here, we further confirmed that miR-138-5p was elevated with age and negatively correlated with osteoblast differentiation and osteogenesis in human tissues, mice and cultured cells (Figure 1). In our previous study, we screened and identified several miRNAs, including miR-138-5p, as potential biomarkers or therapeutic targets in human osteoporosis 14, 34, 35. These data suggested that miR-138-5p might play an important role in senile osteoporosis.

Recently, miR-138-5p was reported to play vital roles in different bone diseases, including osteoarthritis 36, 37, osteosarcoma 38 and osteoporosis 14, 39, 40. Moreover, the functions of miR-138 in the osteogenetic differentiation process attracted broad attention^11^, ^12^, ^39^, ^41^, ^42^ after Eskildsen et al. verified that miR-138 modulates the osteogenic differentiation of human mesenchymal stem cells 40. In addition, we previously found that miR-138-5p negatively regulated osteoblast differentiation and bone formation under different mechanical conditions 14 and simulated microgravity 15. In the present study, we constructed a stable miR-138-5p overexpression osteoblastic cell line (Hi-138) *in vitro* and an osteoblastic miR-138-5p transgenic (TG) mice model* in vivo* to deeply investigate the effects of miR-138-5p gain-of-function on aging-related osteogenesis. A high miR-138-5p level enhanced the decrease in osteoblast differentiation induced by aging (Figure 2). Specifically, the bone formation rate, trabecular microarchitecture and skeletal mechanical strength properties became significantly weaker with age in TG mice, compared to the WT mice (Figure 3). These results demonstrated that elevated miR-138-5p aggravated decrease of osteoblast differentiation and bone formation induced by aging. Given that aging-related osteoporosis is a growing public health concern, it is necessary to identify effective approaches for the treatment of this disease. With regard to translational medicine for therapeutic consideration, a pharmacological miR-138-5p inhibitor alleviated the decrease in bone formation and improved senile bone loss symptoms in aged mice (Figures 6 and 7), suggesting a novel potential therapeutic strategy for aging-related osteoporosis.

Previously, multiple miR-138 target genes during the osteogenic differentiation process have been reported, including *Fak 40, 43, Zeb2* 42*, Eif4ebp1* 44*, BMPR2* 45*, Trps1 and Sulf2* 46*,* but these studies mostly focused on the osteogenic differentiation of MSCs. In our previous study, we found that miR-138-5p could directly target microtubule actin crosslinking factor 1 (MACF1) in primary osteoblast differentiation 14, which was further confirmed here (Figure 4D). After mutation of the binding sequence of miR-138-5p and the *Macf1* 3'UTR, the luciferase activity in osteoblasts was not significantly changed by treatment with agomir-138-5p 14 or antagomir-138-5p in osteoblasts (Figure 4). These data show that MACF1 is a potential target of miR-138-5p in osteoblast differentiation.

MACF1, an important cytoskeletal protein, is crucial in controlling cytoskeleton dynamics through binding to both microtubules and F-actin 47, 48. In our laboratory, we have long concentrated on the roles and mechanisms of MACF1 in different bone cells and various bone diseases 14, 21-23, 49-55. In osteoblasts, MACF1 promotes osteoblast proliferation, migration and differentiation by regulating Wnt/β-catenin signaling 23, 49, 50, 52, 55. Recently, we found that miR-138-5p suppresses osteoblast differentiation by inhibiting β-catenin under simulated microgravity 15. These results provide a possible underlying mechanism for the involvement of miR-138-5p in senile osteoporosis. Furthermore, we established osteoblast-specific MACF1 conditional knockout mice with Osterix promoter to closely examine the function of MACF1 in bone formation *in vivo*. The results showed that deficiency of MACF1 in osteoblasts inhibited osteoblast differentiation and bone formation through the Bmp2/Smad/Runx2 pathway 22. In a recent study, we found that the expression of MACF1 decreased with age in femur tissues of patients with aging-related osteoporosis, and overexpression of MACF1 alleviated the decrease in bone formation and osteoporosis in aged mice 53. In the present study, we also found that the reduced osteoblast differentiation induced by low MACF1 levels was aggravated after etoposide-induced aging (Figure 5) and that miR-138-5p regulated aged osteoblast differentiation depended on MACF1. In addition, the therapeutic treatment of aged mice with a pharmacological miR-138-5p inhibitor led to the significant elevation of MACF1 expression (Figure 7), which indicated that the inhibition of miR-138-5p alleviated senile osteoporosis by targeting the regulation of MACF1.

In summary, the current findings identify a new aging-related miRNA, miR-138-5p, that negatively modulates osteoblast differentiation and bone formation in senile osteoporosis by targeting MACF1. High miR-138-5p levels aggravate the decrease in osteoblast differentiation and severity of bone loss with age. Moreover, treatment with a pharmacological miR-138-5p inhibitor could alleviate aging-related bone loss, providing a potential therapeutic approach for treating senile osteoporosis.

## Supplementary Material

Supplementary figures and tables.

## Abbreviations

ALP/Alp: alkaline phosphatase
agomir: agomir-138-5p
antagomir: antagomiR-138-5p
ARS: alizarin red staining
BMC: bone mineral content
BMD: bone mineral density
BV/TV: bone volume to tissue volume (bone volume fraction)
ColIα1/COLIα1: collagen type 1 alpha-1
GAPDH/Gapdh: glyceraldehyde3-phosphatedehydrogenase
Hi-138: stable miR-138-5p overexpression osteoblastic cell line
Hi-NC: Hi-138 corresponding negative control osteoblastic cell line
IHC: immunohistochemical
KD-Macf1: stable MACF1 low-expressing osteoblastic cell line
KD-NC: KD-Macf1 corresponding negative control osteoblastic cell line
MACF1/Macf1: microtubule actin cross-linking factor 1
MAR: mineral apposition rate
microCT: Micro-computed tomography
miRNA: microRNA
NC: antagomir-NC
OCN: osteocalcin
OB.N/B.Pm: osteoblast number per bone perimeter
WT: Wild type mice
Runx2/RUNX2: runt-related transcription factor 2
Si-Macf1: Macf1 siRNA
Si-NC: negative control siRNA
Tb.N: trabecular number
Tb.Th: trabecular thickness
Tb.Sp: trabecular spacing
TG: osteoblastic miR-138-5p transgenic mice
Veh: osteoblast-targeted delivery system
