# Supplementary material for: MiR-138-5p Targets MACF1 to Aggravate Aging-related Bone Loss
Source: Int J Biol Sci. 2022 Jul 18;18(13):4837–52. doi: 10.7150/ijbs.71411 (PMC9379396; doi:10.7150/ijbs.71411)
Supplement: Supplementary file 1 — Supplementary figures and tables. [file ijbsv18p4837s1.pdf]

SUPPLEMENTARY FIGURES

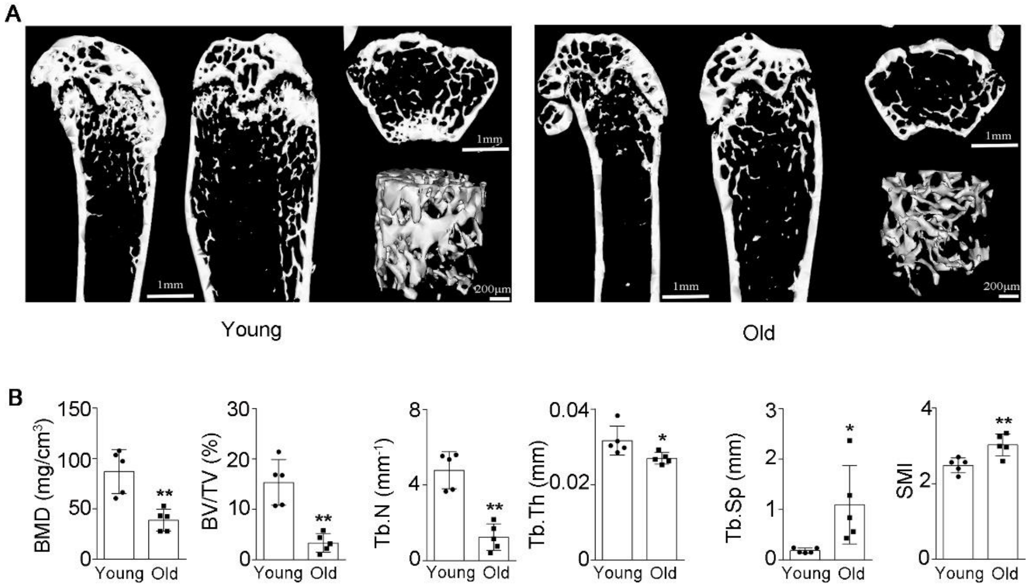

S-Figure 1. Bone loss increases with age. (**A**) Representative 3D reconstruction images showing microarchitecture in distal femur of young (6-month-old) and old (21-month-old) mice. Left and right upper scale bar, 1 mm; right lower scale bar, 200 µm. (**B**) The microCT statistical analysis of BMD, BV/TV, Tb.Th, Tb.N, SMI and Tb.Sp in distal femur of young (6-month-old) and old (21-month-old) mice. BMD, bone mineral density; BV/TV, bone volume to tissue volume; Tb.N, trabecular number; Tb.Th, trabecular thickness; SMI, structure model index; Tb.Sp, trabecular spacing. n = 5 mice for each group. Data are represented as mean ± sd. Significances were determined using student's *t*-test between two groups. *P* value less than 0.05 were considered significant in all cases (**P* < 0.05, ***P* < 0.01).

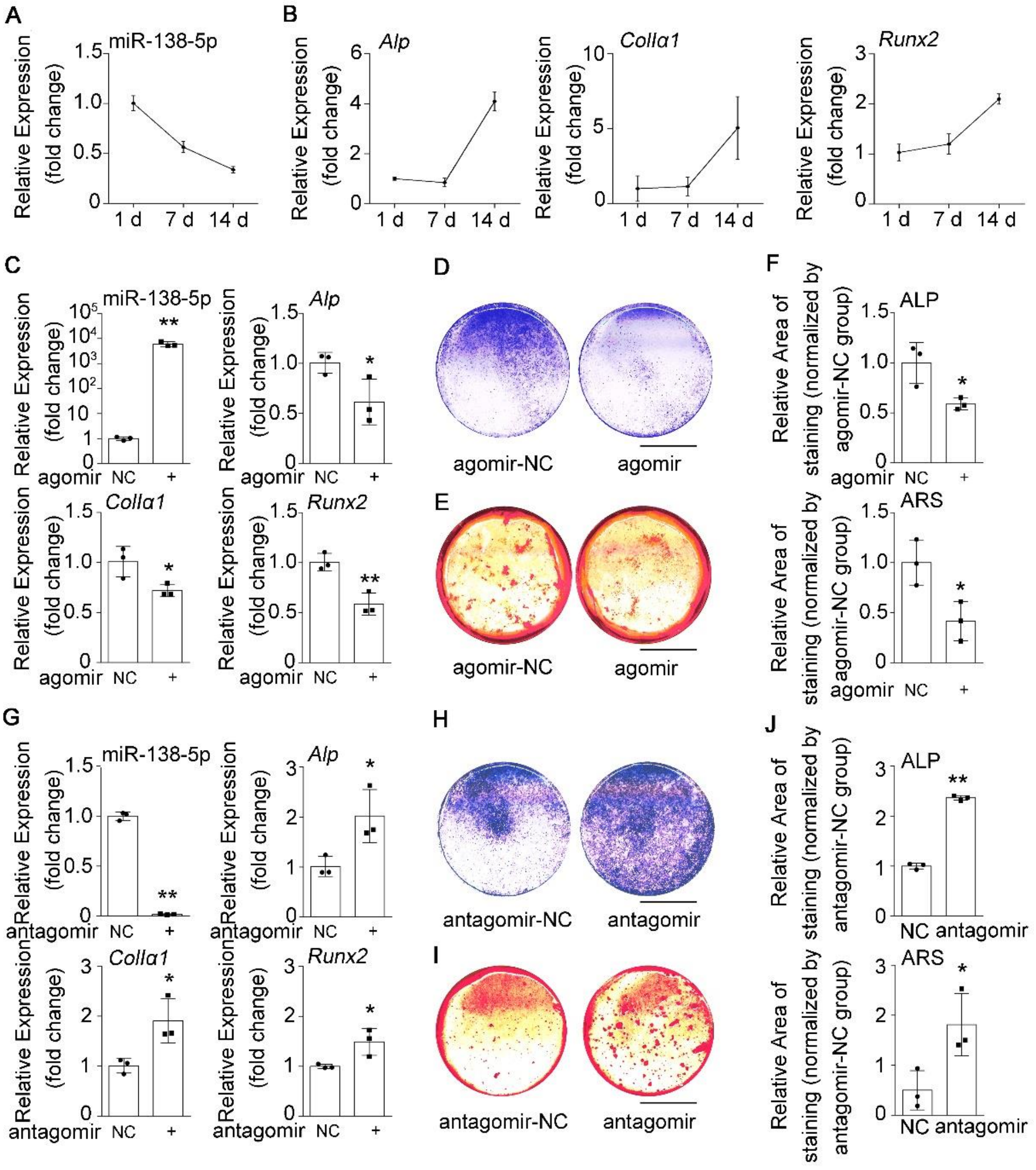

S-Figure 2. miR-138-5p inhibits primary calvarial osteoblast differentiation. (**A**) Real-time PCR analysis of miR-138-5p relative expression level in primary calvarial osteoblasts at different time points (1 day, 7 days, 14 days) in the osteogenic differentiation process. n = 3 for each group. (**B**) Real-time PCR analysis of osteogenic marker gene *Alp, CollIα1* and *Runx2* in primary calvarial osteoblasts at different time points (1-day, 7-day, 14-day) in the osteogenic differentiation process. n = 3 for each group. (**C**) Real-time PCR analysis of miR-138-5p and osteogenic marker gene (*Alp*, *CollIα1* and *Runx2*) in primary calvarial osteoblasts treated with either 50 nM agomir-138-5p (agomir) or agomir-NC for 48 h. n = 3 for each group. (**D**) Representative images of ALP staining in primary calvarial osteoblasts treated with either 50 nM agomir-138-5p (agomir) or agomir-NC for 48 h. Scale bar, 5 mm. (**E**) Representative images of Alizarin red staining (ARS) in primary calvarial osteoblasts treated with either 50 nM agomir-138-5p (agomir) or agomir-NC for 15 d.

Scale bar, 5 mm. (**F**) Quantification of ALP staining areas (upper) and ARS staining areas (lower, 15 d) in primary calvarial osteoblasts treated with either 50 nM agomir-138-5p (agomir) or agomir-NC. n = 3 for each group. (**G**) Real-time PCR analysis of miR-138-5p and osteogenic marker genes (*Alp, ColIα1* and *Runx2*) in primary calvarial osteoblasts treated with either 50 nM antagomir-138-5p (antagomir) or antagomir-NC for 48 h. n = 3 for each group. (**H**) Representative images of ALP staining in primary calvarial osteoblasts treated with either antagomir-138-5p (antagomir) or antagomir-NC for 48 h. Scale bar, 5 mm. (**I**) Representative images of Alizarin red staining (ARS) in primary calvarial osteoblasts treated with either 50 nM antagomir-138-5p (antagomir) or antagomir-NC for 13 d. Scale bar, 5 mm. (**J**) Quantification of ALP staining areas (upper) and ARS staining areas (lower, 13 d) in primary calvarial osteoblasts treated with either 50 nM antagomir-138-5p (antagomir) or antagomir-NC. n = 3 for each group. U6 small nuclear RNA was used as the internal control for miR-138-5p, and *Gapdh* was used as the internal control for mRNAs. Data are represented as mean ± s.d. Significances were determined using student's *t*-test between two groups. *P* value less than 0.05 was considered significant in all cases (*$P < 0.05$, **$P < 0.01$).

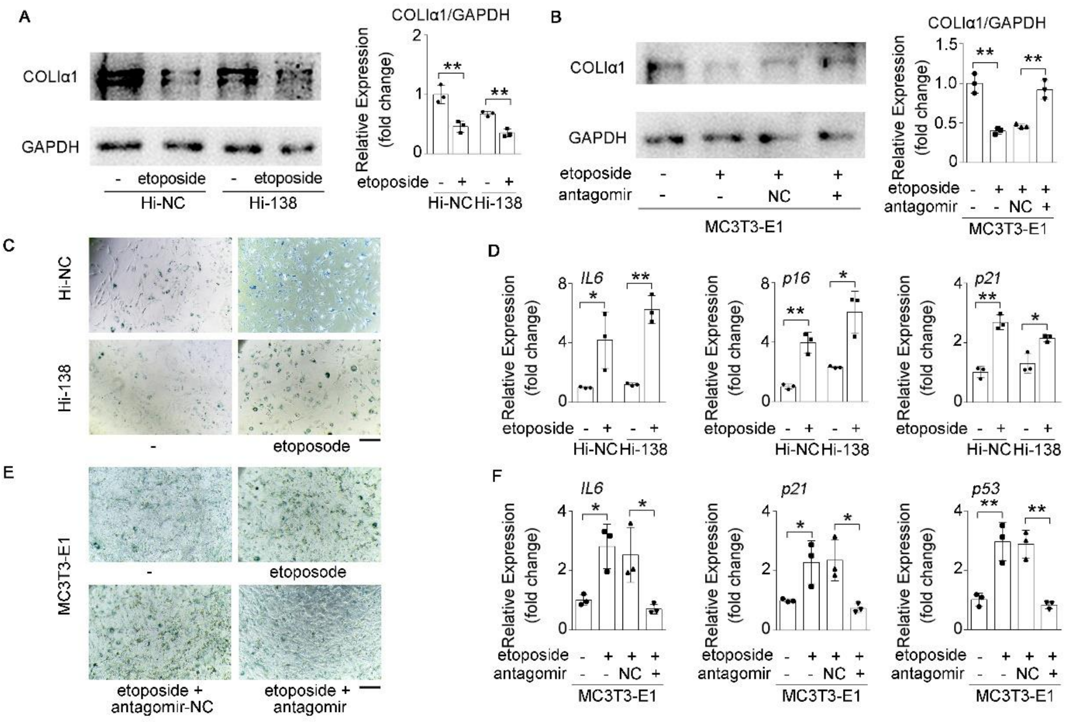

S-Figure 3. High miR-138-5p levels aggravate the decrease in osteoblast differentiation induced by aging. (**A**) Western Blot analysis and quantification of COLIα1 protein level in stable miR-138-5p overexpression osteoblastic cell line (Hi-138) in Hi-138 cells or Hi-NC cells treating with 2-day etoposide-induced aging, respectively. n = 3 for each group. (**B**) Western Blot analysis and quantification of COLIα1 and RUNX2 protein levels in MC3T3-E1 cells treating with either antagomir-138-5p or antagomir-NC after 2-day etoposide-induced aging, respectively. n = 3 for each group. (**C**) Representative images of β-galactosidase staining in Hi-138 cells or Hi-NC cells treating with 2-day etoposide inducing aging, respectively. Scale bar, 100 μm. n = 3 for each group. (**D**) Real-time PCR analysis of senescent marker gene (*IL6*, *p16* and *p21*) mRNA levels in Hi-138 cells or Hi-NC cells treating with 2-day etoposide-induced aging, respectively. n = 3 for each group. (**E**) Representative images of β-galactosidase staining in MC3T3-E1 cells treating with either antagomir-138-5p or antagomir-NC after 2-day etoposide-induced aging for 48 h, respectively. Scale bar, 100 μm. n = 3 for each group. (**F**) Real-time PCR analysis of senescent marker gene (*IL6*, *p21* and *p53*) mRNA levels in MC3T3-E1 cells treating with either antagomir-138-5p or antagomir-NC after 2-day etoposide-induced aging, respectively. n = 3 for each group. *Gapdh* was used as the internal control for mRNAs. Data are represented as mean ± s.d. Two-way ANOVA was performed to study the interaction between two independent variables. Then, statistical differences among three or more groups were analyzed via one-way ANOVA and significances were determined using student's *t*-test between two groups. *P* value less than 0.05 was considered significant in all cases (**P* < 0.05, ***P* < 0.01).

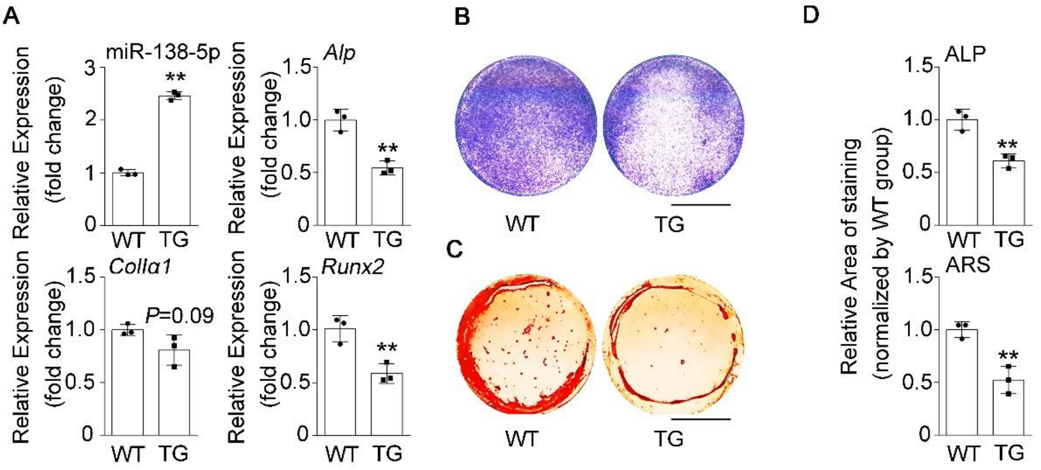

S-Figure 4. High miR-138-5p levels suppresse primary calvarial osteoblast differentiation in osteoblastic miR-138-5p transgenic (TG) mice. (**A**) Real-time PCR analysis of miR-138-5p and osteogenic genes (*Alp, CollαI* and *Runx2*) in primary calvarial osteoblasts isolated from the osteoblastic miR-138-5p transgenic (TG) mice and wild-type (WT) mice. n = 3 for each group. (**B**) Representative images of ALP staining in primary calvarial osteoblasts isolated from TG mice and WT mice. Scale bar, 5 mm. (**C**) Representative images of Alizarin red staining (ARS) in primary calvarial osteoblasts isolated from TG mice and WT mice for 16 d. Scale bar, 5 mm. (**D**) Quantification of ALP staining areas (upper) and ARS staining areas (lower, 16 d) in primary calvarial osteoblasts isolated from TG mice and WT mice for 16 d. n = 3 for each group. U6 small nuclear RNA was used as the internal control for miR-138-5p, and *Gapdh* was used as the internal control for mRNAs. Data are represented as mean ± s.d. Significances were determined using student's *t*-test between two groups. *P* value less than 0.05 was considered significant in all cases (**P* < 0.05, ***P* < 0.01).

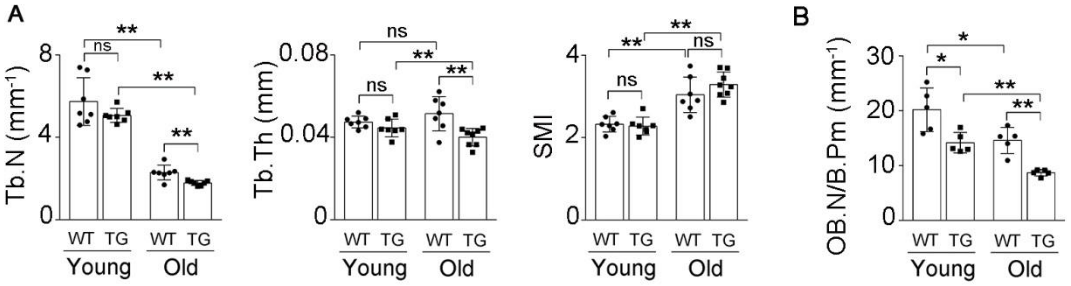

S-Figure 5. High miR-138-5p levels aggravate aging-related trabecular bone loss. (**A**) The microCT statistical analysis of Tb.N,Tb.Th and SMI in distal femur of osteoblastic miR-138-5p transgenic (TG) mice and WT mice, respectively. young, 3-month-old, old, 19-month-old. Tb.N, trabecular number; Tb.Th, trabecular thickness; SMI, structure model index. Young-WT, n = 7; Young-TG, n = 7; Old-WT, n = 7; Old-TG, n = 8. (**B**) Static histomorphometric analysis of OB.N/B.Pm showing osteoblast number in distal femur of TG mice and WT mice, respectively. young, 3-month-old, old, 19-month-old. n = 5 for each group. Data are represented as mean ± sd. Data are represented as mean ± s.d. Two-way ANOVA was performed to study the interaction between two independent variables. Significances were determined using student's *t*-test between two groups. *P* value less than 0.05 was considered significant in all cases. (**P* < 0.05, ***P* < 0.01, ns: no significant difference).

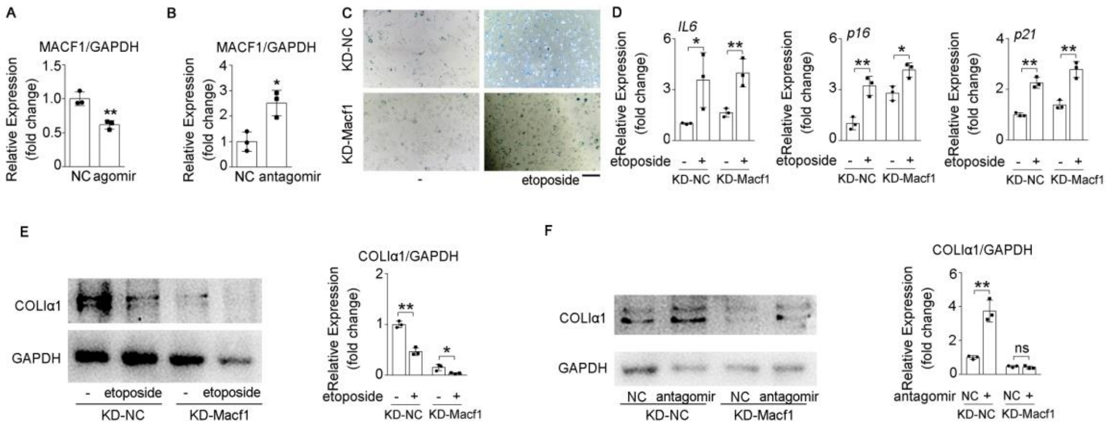

S-Figure 6. High miR-138-5p levels aggravate the decrease in osteoblast differentiation induced by aging. (**A**) Quantification of MACF1 protein level in MC3T3-E1 cells treating with either agomir-138-5p (agomir) or agomir-NC for 48 h, respectively. n = 3 for each group. (**B**) Quantification of MACF1 protein level in MC3T3-E1 cells treating with either antagomir-138-5p (antagomir) or antagomir-NC for 48 h, respectively. n = 3 for each group. (**C**) Representative images β-galactosidase staining in stable MACF1 low-expressing (KD-Macf1) cells treating with 2-day etoposide-induced aging, or in negative control cell line (KD-NC) for 48 h, respectively. Scale bar, 100 μm. n = 3 for each group. (**D**) Real-time PCR analysis of senescent marker gene (*IL6*, *p16* and *p21*) mRNA levels in KD-Macf1 cells or KD-NC cells either treating with 2-day etoposide-induced aging for 48 h, respectively. n = 3 for each group. (**E**) Quantification of COLIα1 protein level in KD-Macf1 cells or KD-NC cells either treating with 2-day etoposide-induced aging for 48 h, respectively. n = 3 for each group. (**F**) Western Blot analysis and quantification of COLIα1 protein level KD-Macf1 cells or KD-NC cells treating with either antagomir-138-5p or antagomir-NC for 48 h, respectively. n = 3 for each group. *Gapdh* was used as the internal control for mRNAs. Data are represented as mean ± s.d. Two-way ANOVA was performed to study the interaction between two independent variables. Then, statistical differences among three or more groups were analyzed via one-way ANOVA and significances were determined using student's *t*-test between two groups. *P* value less than 0.05 was considered significant in all cases (*$P < 0.05$, **$P < 0.01$).

S-Table 1 Clinical features of patients involved in bone specimens analysis

Number	Gender	age (years)	*T*-score for spine	CTX-1 (ng/ml)	PINP (ng/ml)
1	Female	60	-0.2	0.350	48.308
2	Female	61	-0.5	0.371	60.348
3	Female	63	-0.2	0.374	35.635
4	Female	63	-2.5	0.377	33.844
5	Female	65	-2.7	0.448	34.614
6	Female	69	-1	0.450	29.523
7	Female	70	-3	0.560	28.981
8	Female	76	-3.4	0.617	26.698
9	Female	76	-4	0.610	26.368
10	Female	77	-3.1	0.739	23.881
11	Female	77	-3.5	0.661	30.026

S-Table 2 60-69 years group *vs* 70-79 years group

	60-69 ys (n = 6)	70-79 ys (n = 5)
T-score	-1.18 ± 0.46	-3.40 ± 0.18**
CTX-1 (ng/ml)	0.395 ± 0.018	0.637 ± 0.030**
PINP (ng/ml)	40.378 ± 4.754	27.191 ± 1.075*

*$P < 0.05$, **$P < 0.01$
